# Plasma fibroblast skin tightening treatment resulting in bilateral chemical eye injury secondary to EMLA cream: a case report

**DOI:** 10.1186/s12886-020-01613-8

**Published:** 2020-08-24

**Authors:** Sirjhun Patel, Mohith Shamdas, Caroline Cobb

**Affiliations:** 1 Department of Ophthalmology, Ninewells Hospital and Medical School Dundee, Dundee, DD1 9SY UK; 2 Department of Ophthalmology, University of Dundee, Dundee, UK

**Keywords:** Chemical eye injury, EMLA cream, Plasma fibroblast skin tightening, Case report

## Abstract

**Background:**

Plasma fibroblast skin tightening treatment is a relatively novel and growing minimally invasive aesthetic skin procedure. The treatment claims to rejuvenate skin by improving facial lines, wrinkles and skin pigmentation associated with photo-ageing. The skin is often anaesthetised prior to the procedure with topical creams such as EMLA (Eutectic mixture of local anaesthetics). We present the first case of bilateral chemical eye injury following plasma fibroblast skin tightening treatment secondary to EMLA cream. EMLA cream was inadvertently administered to both eyes in preparation for the treatment.

**Case presentation:**

A patient was referred from the emergency department to a tertiary ophthalmology centre with bilateral exquisite eye pain, inability to open the eyes, photosensitivity and reduced vision. She underwent cosmetic plasma fibroblast skin tightening treatment at her local salon four hours earlier. She was found to have bilateral alkali chemical eye injuries with significant diffuse corneal epithelial loss. The injury was thought to be caused by inadvertent ocular exposure to EMLA cream, which was used in preparation for the plasma fibroblast skin tightening treatment. She was treated with topical antibiotics and achieved satisfactory recovery.

**Conclusion:**

This case report highlights a possible complication following plasma fibroblast skin tightening treatment. We lay emphasis on the importance identifying chemical injury and recommend that medication attention should be sought if there is any concern.

## Background

Demand for minimally-invasive aesthetic skin procedures has spawned a number of treatment modalities for skin regeneration and its rejuvenation. Touted as a technique for ‘non-surgical blepharoplasty’, plasma skin regeneration is gaining popularity as a high-street aesthetic procedure. This non-invasive procedure is especially used in the treatment of mild upper dermatochalasis and lower eyelid lines and wrinkles. It is also known as ‘fibroblast skin tightening’ or ‘plasma pen’. The exact mechanism of its action is debated. An ultra-high-frequency generator ionises inert atmospheric nitrogen into an active plasma that delivers controlled thermal energy to the skin via a handpiece [1]. The thermal energy is believed to remove old photodamaged epidermal cells, and stimulate fibroblast activity and collagen growth within the upper dermis [2]. Temporary hyperpigmentation is often reported in the immediate post-procedural period, particularly with higher energy level treatments [3]. The skin is commonly prepared with a topical anaesthetic creams such as EMLA (Eutectic mixture of local anaesthetics).

Lidocaine with prilocaine 5% cream is commonly known by the trade name EMLA cream, which is an abbreviation for ‘eutectic mixture of local anaesthetics’. Each gram of cream contains 25 mg lidocaine, 25 mg prilocaine, macrogolglycerol hydroxystearate, carbopol 974P, sodium hydroxide and purified water [4]. It is local anaethtic cream that is used commonly to anaesthetise the skin. Sodium hydroxide gives EMLA cream an alkaline pH of 9, which allows for penetration of anaesthetic agents. It is currently available to buy without a prescription from pharmacies in the UK. Alkali agents are lipophilic and penetrate the eye more rapidly than acids. Alkali chemicals penetrate cell membranes through saponification and denature the collagen matrix of the cornea [5]. The damaged tissues can undertake liquefaction necrosis and secreted proteolytic enzymes which can lead to a cascade of further damage.

EMLA cream is very well known to cause corneal chemical alkaline burns from inadvertent exposure [6, 7]. Children have shown features of alkali eye injury from EMLA cream after small amounts of the substance has been self-applied accidently [8].

This case report describes the first case of bilateral chemical eye injury caused form the plasma fibroblast skin tightening procedure. EMLA cream was inadvertently administered to both eyes in preparation for treatment.

## Case presentation

A 60 year old caucasian female underwent cosmetic fibroblast skin tightening treatment around both her eyelids at her local salon. She recalls some of the EMLA cream (used as a topical anaesthetic) inadvertently seeping into both her eyes during preparation for the treatment. She experienced some initial discomfort, but recalls it subsided and proceeded to have the fibroblast treatment.

She presented approximately four hours later to the emergency department with acute, bilateral, red, painful, photophobic eyes with reduced vision and periorbital swelling. She was referred as an emergency to ophthalmology with bilateral corneal injuries.

Her visual acuity on initial examination was 6/12 right and 6/24 left. Examination was very difficult as she was extremely photophobic and rated the pain as 10/10 on the pain scale. pH was 7–8 in both eyes measured with litmus paper.

**Fig. 1:**
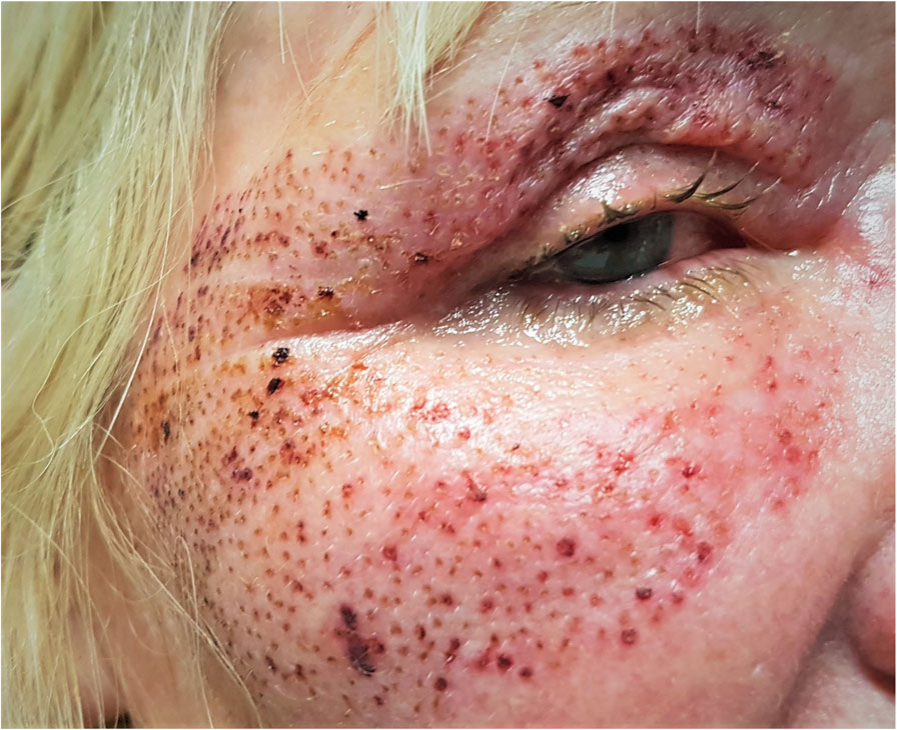
Bilateral periorbital oedema with multiple dot burn lesions on her skin secondary to the plasma fibroblast skin tightening treatment

**Fig. 2:**
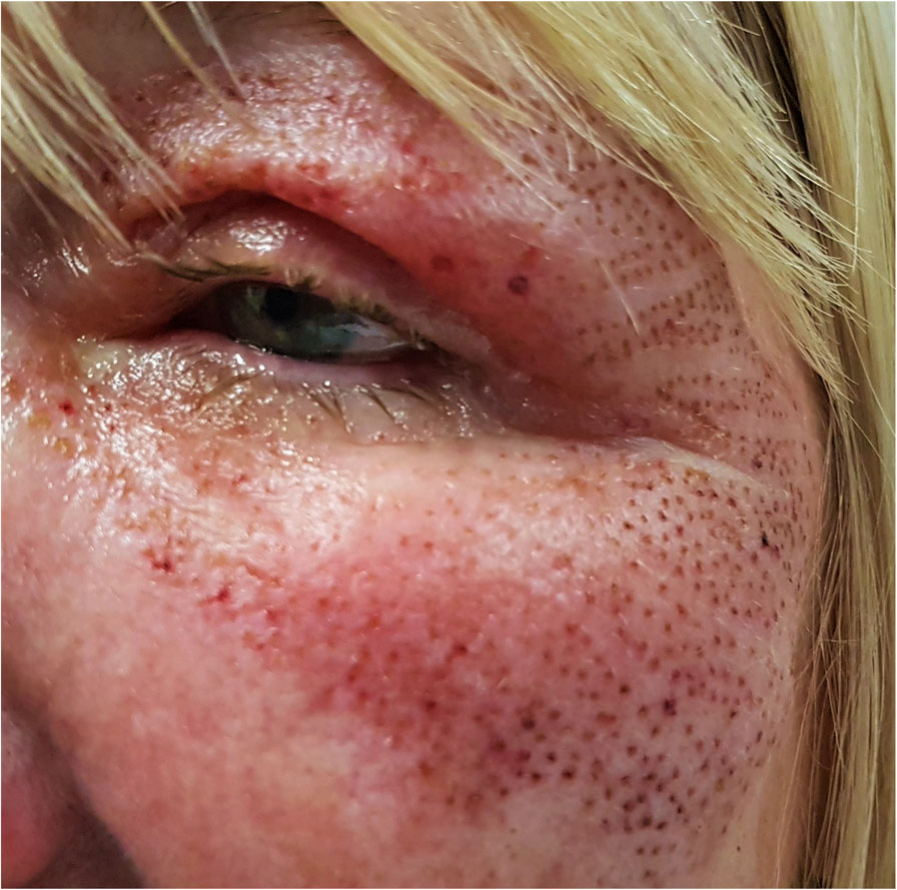
Bilateral periorbital oedema with multiple dot burn lesions on her skin secondary to the plasma fibroblast skin tightening treatment

On examination she was found to have bilateral diffuse corneal epithelial loss affecting approximately 80% of both corneas. These findings were consistent with a bilateral corneal chemical alkaline injury. She also displayed marked periorbital oedema with multiple dot burn lesions on her skin secondary to the plasma fibroblast treatment itself (Figs. 1 and 2).

The patient was treated with topical chloramphenicol 1% ointment every 2 h for both eyes, topical cyclopentolate 1% and wore soft eye pads over her eyes overnight for comfort. After day 1 of treatment, she was switched to preservative free chloramphenicol 0.5% eye drops 4 times a day to both eyes and chloramphenicol ointment once at night.

On day 5, the patient was prescribed preservative-free sodium hyaluronate 0.2% lubricating eye drops for use during the day and a paraffin-based ointment for use at night.

**Fig. 3:**
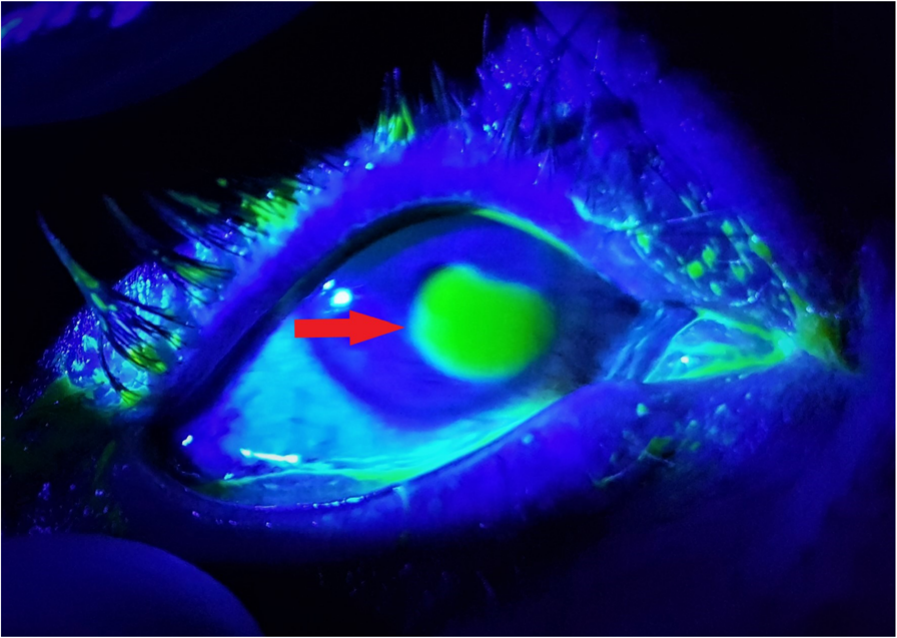
Anterior segment view of the right eye illuminated with a cobalt blue filter. Red arrow: A large 4.3 mm × 6 mm corneal epithelial defect visible with 1% fluorescein

The patient had a visual acuity was 6/18 in both eyes on review the following day. The patient was less photophobic and fluorescein examination showed bilateral resolving epithelial defects. After day 1 of treatment the right eye still had a large epithelial defect (Fig. 3). Figure 3 shows a large 4.3 mm by 6 mm epithelial defect in the right eye with 1% fluorescein illuminated with a cobalt blue filter.

On day 5 follow up, the corneal epithelial defects were mostly resolved with only a few diffuse punctate epithelial erosions seen with fluorescein in both eyes. The patient reported symptoms of dry eye and mild photophobia. Visual acuity returned to 6/6 in both eyes.

**Fig. 4:**
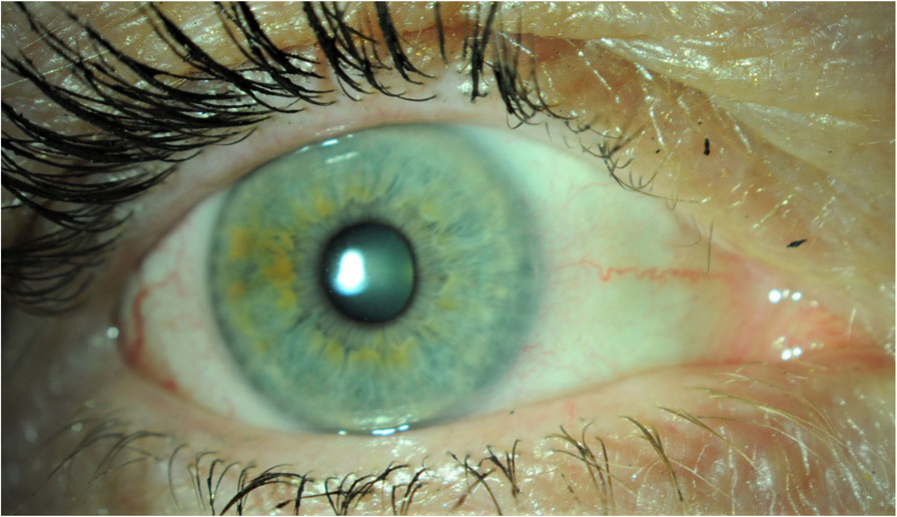
Pictures of the patients right and left ocular surface on day 25 of follow up

**Fig. 5:**
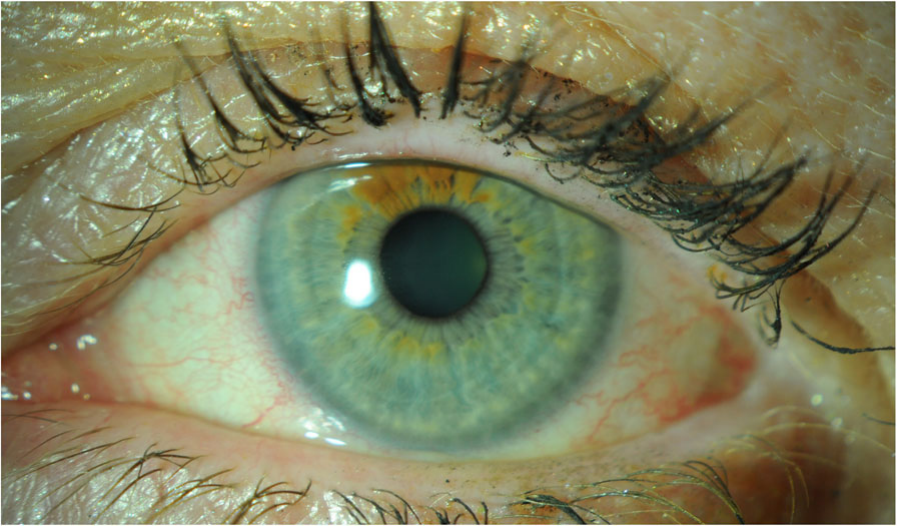
Pictures of the patients right and left ocular surface on day 25 of follow up

On day 25 of follow up the patient reported only very mild dry eye symptoms and the ocular surface had returned to normal in both eyes (Figs. 4 and 5). The patient was advised that she is at risk of developing bilateral corneal recurrent erosion syndrome.

## Discussion and conclusions

From this case we can infer that the patient sustained a chemical eye injury secondary to inadvertent EMLA cream instillation to the eyes in preparation for her plasma fibroblast skin tightening treatment.

This case exhibits a delayed presentation of pain. The patient felt an initial irritation from the EMLA cream but this later subsided. She presented with painful eyes approximately four hours after her treatment at the salon. This is due to the anaesthetic effect of EMLA cream on the ocular surface. This masking property of topical anaesthetics makes it dangerous if not used appropriately. There is a case report of a patient presenting with eye symptoms as delayed as the following day after inadvertent exposure to EMLA cream during skin treatment [9].

In this case, the pain settling proved to be inappropriately reassuring for both the patient and the practitioner administering the skin treatment. Immediate ocular surface irrigation was not commenced at the salon. Rather, the occlusive effect of the patient closing her eyes may have resulted in retention of EMLA cream over the ocular surface for the duration of the treatment. We can assume that the chemical injury to the ocular surface was intensified because of this. The practitioner administering the local anaesthetic claimed that she was unaware of the nature of EMLA cream and that this information was not included in her training. Considerable caution should be taken when applying EMLA cream to the periocular skin as there is a risk of migration of the anaesthetic to the ocular surface. There is also a possibility of drug permeation through the skin of the eyelid which could result in drug delivery to the conjunctiva and other ocular tissues [10].

If EMLA cream comes into contact with the ocular surface it is recommended to immediately irrigate the eye with luke warm water or salt solution [4]. As topical anaesthetics on the ocular surface can initially mask symptoms – we recommend abandoning any skin procedures until an appropriate assessment has been made and sensation returns. If the eye is showing any signs of irritation we recommend a low threshold to seek advice from a trained medical clinician. Urgent attention should be sought if there is any concern of chemical eye injury. pH should be checked and copious irrigation commenced without delay.

## Data Availability

Not applicable.

## Abbreviation

EMLA: Eutectic mixture of local anaesthetics
